# Simpson's Paradox of social media opinion's response to COVID-19

**DOI:** 10.3389/fpubh.2025.1448811

**Published:** 2025-02-27

**Authors:** Qing Liu, Hosung Son

**Affiliations:** ^1^Pukyong National University, Busan, Republic of Korea; ^2^School of Economics and Management, Huainan Normal University, Huainan, China

**Keywords:** Simpson's Paradox, COVID-19, epidemic intensity, social media opinion, public emotional responses

## 1 Introduction

Simpson's Paradox is a statistical phenomenon where trends observed in subgroups contradict those seen in the overall dataset (1). When data is segmented for analysis, the direction of relationships can reverse, highlighting the complexities of interpreting subgroup-level patterns (2). This paradox underscores the importance of rigorous analysis in complex datasets, as it may obscure causal relationships or lead to erroneous conclusions (1, 3). Its implications extend to various probability-dependent disciplines, including decision theory, causal inference, and evolutionary biology (4, 5).

While not intentionally sought, the COVID-19 pandemic has indeed brought about academic prosperity in the fields of medicine and statistics (6–8). Within these studies, there is no shortage of discoveries related to Simpson's Paradox, emphasizing the crucial importance of understanding this paradox for drawing accurate conclusions from data (9). Similarly, Rasool et al. (10), Hong et al. (11), and Han et al. (12) highlight the utility of advanced correlation methods and data-driven approaches in analyzing public sentiment, financial responses, and content recommendation during crises.

During the COVID-19 pandemic, instances of Simpson's Paradox, while not widespread, were observed in several studies, often highlighting challenges in interpreting complex datasets. For example, Sy (13) confirmed the presence of Simpson's Paradox in correlations between excess mortality and COVID-19 injections, suggesting potential unreliability in such findings. Shaki (14) identified similar issues in the World Health Organization's (WHO) early mortality rate predictions, where statistical methods exhibited Simpson's Paradox, leading to initially overestimated fatality rates that later proved lower. These cases underscore the importance of careful statistical analysis in high-stakes public health contexts.

Further illustrating this phenomenon, Raoult (15) demonstrated how mixing patient data with varying stages of illness, treatment dosages, and durations in studies on hydroxychloroquine efficacy produced erroneous results attributable to Simpson's Paradox. Similarly, Lu (16) emphasized the need for caution in revealing causal relationships in COVID-19 case statistics, using the paradox as a framework to critique oversimplified data interpretations. These examples collectively highlight how Simpson's Paradox can obscure critical insights, particularly during crises where decisions hinge on accurate data analysis.

During the COVID-19 pandemic, the study of public psychology and opinion dynamics has gained significant attention. While Simpson's Paradox has been extensively discussed in medical research and statistical case studies (17, 18), its application to public opinion remains largely unexplored. This study bridges that gap by extending the concept of Simpson's Paradox to analyze public sentiment, highlighting how aggregated data can obscure subgroup-specific trends in emotional responses to crises. By focusing on lagged correlations between epidemic intensity and news volume, our approach captures the temporal complexities of public opinion that static analyses overlook. This research not only deepens understanding of individual and societal psychological responses but also introduces a novel perspective on the dynamic interplay between public sentiment and external stimuli during crises significance (6, 19, 20).

## 2 The paradox of COVID-19 intensity and news volume correlation

### 2.1 Data description

The extensive transmission of COVID-19 in China commenced in January 2020 (21). The formal reclassification of COVID-19 by the Chinese National Health Commission from a “Class B, Level A” to a “Class B, Level B” on 8 January 2023,^1^ may be construed as signifying the conclusion of COVID-19 in China. In a recent scholarly survey, we examined whether fluctuations in COVID-19 intensity during the COVID-19 pandemic in China affect changes in news volume.

For this study, we used COVID-19 new cases and new deaths data from the World Health Organization's data center^2^ as indicators of COVID-19 intensity. To analyze online sentiments, we collected news and comment data from Weitoutiao,^3^ a self-media platform under the banner of “Today's Headlines.” Given the impracticality of collecting all available news texts, we focused on aggregating content from opinion leaders—news media and self-media accounts with an average following exceeding six million—as proxies for public opinion. The data covered the period from 1 January 2020, to 31 December 2022, with statistical intervals spanning 3 days to balance granularity and noise reduction.

Although our study primarily relies on data from a single platform, Weitoutiao, the issue of platform-specific limitations has been significantly mitigated in the era of social media. Opinion leaders and news organizations frequently disseminate their content simultaneously across multiple platforms, such as Sina Weibo, Twitter, and Facebook, to maximize reach and influence. By focusing on opinion leaders with significant influence, our dataset captures trends that extend beyond Weitoutiao, reflecting an interconnected ecosystem of platforms.

The COVID-19 intensity data from 2020 to 2022 can be represented as:


(1) $$\begin{array}{l} CI=\left\{\left(nc_{i},nd_{i}\right)|\;i=1,2,\cdots \;,1096\right\}, \end{array}$$

where (*nc*_*i*_, *nd*_*i*_) represents the daily new cases and new deaths on the i-th day. The set of daily news volumes from 2020 to 2022 can be described as:


(2) $$\begin{array}{l} N=\left\{n_{i}|\;i=1,2,\cdots \;,1096\right\}, \end{array}$$

where *n*_*i*_ denotes the news volume on the i-th day.

To address the issue of data discreteness, we reorganized the above data into statistical intervals of 3 days each. At this point:


(3) $$\begin{array}{l} CI^{3}=\left\{\left(\overset{t^{*}3}{\underset{k={\left(t-1\right)}^{*}3+1}{\sum }}nc_{k},\overset{t^{*}3}{\underset{k={\left(t-1\right)}^{*}3+1}{\sum }}nd_{k}\right)\;|\;t=1,2,\cdots ,365\right\}\; \end{array}$$

and


(4) $$\begin{array}{l} N^{3}=\left\{\overset{t^{*}3}{\underset{k={\left(t-1\right)}^{*}3+1}{\sum }}n_{k}|\;t=1,2,\cdots ,365\right\}. \end{array}$$

The selected 3-day interval also aligns with temporal patterns observed in public opinion studies, where short-term fluctuations tend to stabilize over a few days, allowing for clearer analysis of trends without losing responsiveness to dynamic changes.

### 2.2 Simpson's paradox of correlation

Table 1 illustrates the correlation statistics between the intensity of COVID-19 and the amount of news text in the statistical interval. The correlation between COVID-19 intensity in different years and news volume at different lags can be observed.

**Table 1:** Statistics of followers and news.

**Lag**	**2020**		**2021**		**2022**		**Years 2020–2022**	
	**New cases**	**New deaths**	**New cases**	**New deaths**	**New cases**	**New deaths**	**New cases**	**New deaths**
0	0.0081	0.1692	0.1603	0.1508	0.0081	0.0636	−0.0959	−0.1045^*^
1	0.0229	0.2268^*^	0.1273	0.1211	0.0229	0.0127	−0.0815	−0.1072^*^
2	0.0764	0.1225	0.1032	0.1027	0.0764	0.1846	−0.0767	−0.1312^*^
3	0.1352	0.2686^**^	0.0676	0.0899	0.1352	0.0033	−0.0779	−0.0982
4	0.2018^*^	0.2099^*^	0.0404	0.1138	0.2018^*^	0.0231	−0.0919	−0.0948
5	0.2661^**^	0.1658	0.2249^*^	0.2169^*^	0.2661^**^	0.0753	−0.1192^*^	−0.1037^*^
6	0.2364^*^	0.3364^***^	0.245^**^	0.2123^*^	0.2364^*^	0.0002	−0.1699^**^	−0.0425
7	0.3031^***^	0.3057^***^	0.2275^*^	0.2039^*^	0.3031^***^	0.0009	−0.2396^*****^	−0.0477
8	0.3152^***^	0.3805^*****^	0.2303^*^	0.1706	0.3152^***^	0.0000	−0.236^*****^	−0.0259
9	0.3899^*****^	0.3168^***^	0.21^*^	0.1587	0.3899^*****^	0.0007	−0.2235^*****^	−0.0410
10	0.4099^*****^	0.3572^****^	0.2375^*^	0.2513^*^	0.4099^*****^	0.0001	−0.2319^*****^	−0.0448
11	0.5118^*****^	0.3417^***^	0.25^*^	0.2102	0.5118^*****^	0.0003	−0.2189^*****^	−0.0363
12	0.5090^*****^	0.3546^***^	0.3052^*^	0.2449^*^	0.509^*****^	0.0002	−0.2183^*****^	−0.0433
13	0.5127^*****^	0.3262^***^	0.3548^**^	0.1793	0.5127^*****^	0.0006	−0.2112^****^	−0.0388
14	0.5578^*****^	0.3306^***^	0.4109^***^	0.1138	0.5578^*****^	0.0005	−0.2065^****^	−0.0411
15	0.4939^*****^	0.3389^***^	0.3735^**^	0.0851	0.4939^*****^	0.0004	−0.2092^****^	−0.0320
16	0.5595^*****^	0.3084^**^	0.3615^**^	0.0464	0.5595^*****^	0.0014	−0.1936^***^	−0.0354
17	0.4578^*****^	0.3899^*****^	0.311^*^	0.0247	0.4578^*****^	0.0000	−0.201^***^	−0.0132
18	0.5081^*****^	0.3266^***^	0.0244	0.1138	0.5081^*****^	0.0008	−0.1896^***^	−0.0244
19	0.4771^*****^	0.3296^***^	0.0227	0.1187	0.4771^*****^	0.0007	−0.1937^***^	−0.0305
20	0.434^*****^	0.3002^**^	0.1138	0.1144	0.434^*****^	0.0023	−0.1922^***^	−0.0232
21	0.4268^*****^	0.2028^*^	0.0104	0.1258	0.4268^*****^	0.0430	−0.1847^***^	−0.0545
22	0.3734^****^	0.2000^*^	0.0193	0.1145	0.3734^****^	0.0471	−0.1899^***^	−0.0471
23	0.3480^***^	0.0852	0.0339	0.0963	0.348^***^	0.4045	−0.1754^**^	−0.0784
24	0.2295^*^	0.1856	0.0476	0.0791	0.2295^*^	0.0687	−0.1862^***^	−0.0498
25	0.2528^*^	0.1324	0.0876	0.0046	0.2528^*^	0.1984	−0.1754^**^	−0.0612
26	0.1636	0.1085	0.0832	−0.0628	0.1636	0.2953	−0.1769^**^	−0.0810
27	0.0865	0.1574	0.0749	−0.0945	0.0865	0.1298	−0.1765^**^	−0.0682
28	0.1849	0.1235	0.0695	−0.1758	0.1849	0.2383	−0.1686^**^	−0.0842
29	0.1055	0.1390	0.0238	−0.2524^*^	0.1055	0.1863	−0.1774^**^	−0.0827
30	0.1408	−0.0004	−0.0638	−0.3168^**^	0.1408	0.9966	−0.1625^**^	−0.1136^*^

*P*-values indicate the statistical significance of Pearson correlation coefficients. When *p* > 0.05, the correlation is considered non-significant. The symbols ^*^, ^**^, ^***^, ^****^, and ^*****^ correspond to significance levels of 0.05, 0.01, 0.001, 0.0001, and 0.00001, respectively.

**Figure 1:**
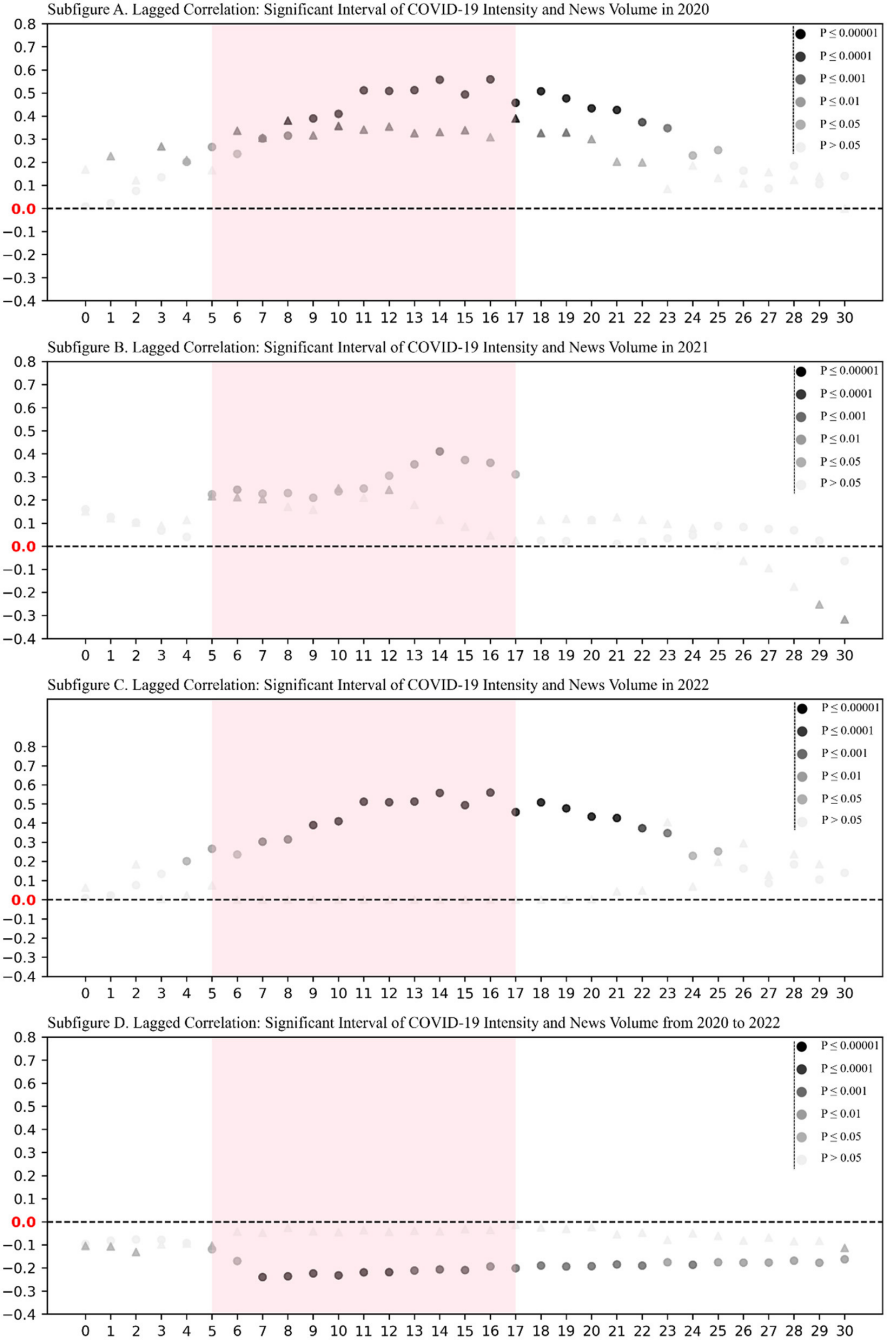
Significant plots of correlation between COVID-19 intensity and news text volume in China. The dashed horizontal line (*y* = 0) provides a reference for distinguishing positive and negative correlations. The figure was created using consistent styling across subplots to highlight key lag ranges and correlation trends. Subplots **(A–C)** reveal strong positive correlations (lags 5–17) in individual years, while subplot **(D)** illustrates a reversal in the aggregated dataset, showcasing Simpson's Paradox.

The complex table data drowns out the statistical laws. In order to observe the statistical laws of the data more clearly, we plotted Figure 1 based on the data in Table 1. In Figure 1, the *x*-axis denotes the lag of news, where, for a given x, we computed the correlation between *CI*^3^ (*i* ∈[0:−*x*] ) and *N*^3^ (*i* ∈[*x*:365]). The color depth of the scatter points indicates the significance of the *p*-value, with darker colors denoting higher levels of significance, as indicated in the figure's legend. The *y*-axis in Figure 1 represents the correlation coefficients, while the origin and triangles signify the significance of new cases and new deaths in relation to news volume, respectively.

Figure 1 illustrates the Simpson's Paradox observed in the correlation between COVID-19 intensity and news volume. In subplots A, B, and C of Figure 1, there is a notable positive correlation between COVID-19 new cases in 2020–2022, and the volume of texts lagged from 5 to 17 periods. This implies that stronger instances of COVID-19 lead to greater publication of news texts. However, as shown in subplot D for the entire 3-year period from 2020 to 2022, there is a significant negative correlation between COVID-19 new cases and lagged text volume from 5 to 17 periods. We treat the data for 2020–2022 as subpopulations and the entire dataset as a whole. The initially observed positive correlation between COVID-19 intensity and news volume during the subperiods undergoes a reversal in the comprehensive analysis.

In sociological research, the volume of news itself is considered a manifestation of emotion (22). The correlation between COVID-19 and news volume reflects people's sentiment toward COVID-19. In our case, the reasons for the emergence of Simpson's Paradox may be multifaceted, such as changes in external conditions during different time periods. From a mathematical and probability perspective, there is no issue with the cases demonstrating this paradox, but the conclusions still leave us surprised.

## 3 Concluding remarks

In this article, we present the Simpson's Paradox observed in the correlation between COVID-19 intensity and news volume during the COVID-19 period in China, both in segmented and holistic analyses. This discovery supplements existing research on Simpson's Paradox related to COVID-19, simultaneously illustrating the complexity of public emotional responses to COVID-19 intensity. Our findings align entirely with the typical Simpson's Paradox mentioned by Sprenger and Weinberger (9), emphasizing that two variables may exhibit a negative correlation in the overall dataset but can be independent or even positively correlated within all subgroups.

The Simpson's Paradox, originally introduced by Simpson (23) and later known as Simpson's Paradox (3), reversal paradox (24), and amalgamation paradox (25), continues to pose challenges to our assessments of causality and our understanding of data, even in the era of artificial intelligence and big data (26, 27). As a statistical phenomenon, Simpson's Paradox emphasizes certain challenges in statistical inference. Accurately comprehending the overall trends in data and the relationships between subgroups is crucial for formulating policies and making well-informed decisions (4, 5).

All fields relying on probability are susceptible to Simpson's Paradox (4, 5). During the COVID-19 period, research mentioning the Simpson's Paradox has primarily been in the domains of medicine and case statistics (13, 16, 28). This study proposes the existence of a Simpson paradox between public opinion and epidemic intensity during the COVID-19 pandemic. This finding not only offers a new perspective for understanding the complexity of opinion formation but also underscores the intricacy of public psychology. However, the analysis was limited to data from the COVID-19 pandemic due to the lack of sufficient long-term public opinion data from other global crises, such as H1N1 or SARS, making broader validation a challenge.

Although this study does not delve into the specific reasons behind the Simpson paradox phenomenon, our findings provide new insights for opinion research and social psychology. Behind the paradoxes we mention, there may be complex emotional dynamics at play, suggesting that exploring the underlying causes could be an intriguing new research topic. Moreover, understanding these paradoxes offers practical value for managing public opinion on social media platforms. By identifying how public sentiment evolves during crises, this research could inform strategies to mitigate misinformation and foster accurate information dissemination, contributing to more effective crisis communication and public sentiment management.

## Footnotes

^1^ https://m.gmw.cn/baijia/2022-12/27/1303236220.html

^2^ https://data.who.int/dashboards/covid19/cases

^3^ https://mp.toutiao.com/docs/mp/2286/16816
